# Talking control sessions in people with advanced cancer: a qualitative analysis of sessions

**DOI:** 10.1186/s40359-022-00837-1

**Published:** 2022-05-20

**Authors:** Daphne Lamirel, Sarah Davis, Joe Low, Marc Serfaty, Megan Armstrong

**Affiliations:** 1 Division of Psychiatry, University College London, 149 Tottenham Court RD, London, W1T 7NF UK; 2 Primary Care and Population Health, University College London, Royal Free Campus, Rowland Hill St, London, NW3 2PF UK

**Keywords:** Advanced cancer, Control group, Talking control, Psychotherapy, Qualitative

## Abstract

**Background:**

Talking control (TC) was developed to control for the common factors in therapy so that the specific effects of therapy can be tested. A TC was recently used in a pilot study of Acceptance and Commitment therapy for dysfunction in people with advanced cancer. This work explores the audio recording of the sessions in a TC to explore how they were utilised by people with advanced cancer.

**Methods:**

This is a qualitative study nested in larger feasibility randomised control trial. The trial participants were recruited from three London hospices. The study examined data for 5 participants who received weekly sessions of a TC. Fifteen sessions, three per participant, were transcribed and analysed using a thematic approach.

**Results:**

Individuals with advanced cancer used TC sessions as a safe place in which they could express their feelings—from smaller daily concerns to deeper-rooted difficulties. Many participants also engaged in emotional and cognitive avoidance regarding some topics, particularly those pertaining to their cancer. The TC sessions were also used as an opportunity to focus on the more positive aspects of their lives. Lastly, they served to reflect on ways to overcome difficulties.

**Conclusions:**

This study suggests the TC can have beneficial, albeit varying uses for people with advanced cancer, that may even be considered therapeutic.

## Background

People with advanced cancer (i.e., not amendable to cure) often face significant psychological distress, due to poor prognosis and diminished quality of life [1, 2]. It is estimated that 17% of participants with advanced cancer experience symptoms of clinical depression [3]. Depression in individuals with cancer has been associated with increased physical symptoms, and when left untreated, an increased likelihood of early death [4]. It is thus recommended by NICE that people with advanced cancer receive adequate psychological treatment [5].

Psychotherapies such as cognitive behavioural therapy (CBT) and acceptance and commitment therapy (ACT) have shown promise across a wide range of disorders [6, 7] including advanced cancer [8, 9]. To test effectiveness psychotherapies should be compared with a control group [10] such as treatment as usual (TAU). Safer and Hugo suggest that to correctly estimate the active ingredient that is specific to a certain form of therapy, one must control for the effects that are common to the outcomes of all types of therapy [11]. Safer and Hugo outline seven steps in which to achieve this including the development of a credible comparison control that is matched up in terms on common factors and then to test that the control sufficiently addresses these common factors and not the specific therapeutic factors. There are over 89 factors that are known to predict outcome which are not believed to be unique to the therapeutic intervention [12, 13]. These include, length and structure of sessions, client and therapist expectations, the establishment of a therapeutic relationship, the ventilation of feelings, the instillation of hope, and the provision of warmth and empathy [14].

A talking control (TC) was used to control for the common effects of therapies, such as ACT or CBT, while maintaining the specific effects of therapy [15, 16]. This TC was originally developed in a pilot study evaluating the effect of CBT in 33 older individuals that were randomized to either TC, treatment as usual and TC, or treatment as usual and CBT [15–17]. The TC is manualised (available from the authors) and involves a conversation led by the participant, whilst the therapist is empathic and validates the patients feelings, without commenting or offering advice or use techniques such as problem solving, challenge beliefs, mindfulness, or explore different ways of behaving. Therapists instead steer the conversation toward topics such as daily activities, television programmes, health, religion, or history. TC was found to be acceptable as a control and identified as useful in 70% of participants [15, 16].

As TC is relatively new, there has been no analysis of the content of the sessions. People with advanced cancer, and with other long-term conditions, often have unmet psychological needs [18] and therefore it is important to explore what people talk about when they are given the opportunity to speak freely. In addition, as TC is used as an active control against non-specific therapeutic effects, it is important to ensure that TC itself does not elicit therapeutic effects and if it does understand the underlying mechanisms.

## Methods

This qualitative descriptive study uses the naturalistic data collected from the TC sessions from the CanACT study [9].

### Aims

To explore the contents of Talking Control sessions for people with advanced cancer in order to understand the underlying mechanisms, to ensure it is an appropriate active control and identify the main topics that participants discuss when given the option to speak freely.

### Ethics

Data used from the CanACT study and methods used in the present study were approved by the Riverside Research Ethics Committee (ref 13/LO/0813) on 4th July 2014 [9]. ISRCTN13841211 (registered 22nd July 2015). All participants gave written informed consent including consent for their intervention transcripts to be analysed. All methods from the CanACT and present study were performed in accordance with relevant guidelines and regulations.

### Setting

The CanACT study took place in London, UK. Participants were recruited from three hospices, in which they were attending palliative care day services either as in- or out-patients.

### Participants

Individuals were selected for participation in the CanACT study, to be randomised to ACT or TC, if (1) they were aged 18 years or more and had a score of less than 81 on the FACT-G score (where lower scores indicate lower functioning) [9] (2) had a diagnosis of advanced cancer not amenable to cure (3) with an estimated survival of 4 months or more according to an expert clinician. Reasons for exclusions included (1) an insufficient command of English, judged by the staff (2) cognitive impairment, and (3) the participants were currently receiving ACT or CBT.

This study’s sample consists of a sub-sample of the TC arm of the CanACT study’s participants (n = 22). Of these, 15 participants completed seven or eight sessions and were deemed to have fully engaged in the intervention. Five individuals were selected at random from the 15 participants who had seven or more sessions.. For each participant, three sessions out of eight were selected—this included the first, middle and last session—to allow for an in-depth analysis of the sessions’ use for each participant. This yielded a total sample size of 15 sessions.

### Procedure

Eligible participants were identified by day-therapy staff between November 2015 to 2016. Consent to participate was obtained in written form. Participants were randomised to eight sessions of ACT plus treatment as usual (TAU) or TC plus TAU. Details of TC can be found in Table 1. All sessions were audio recorded. To ensure this qualitative study had sufficient rigour, we drew upon the ‘Quality Indicators of Rigor in Qualitative Research’ [19].

**Table 1:** Techniques in talking control to be used and avoided

*Techniques to be utilised within Talking Control sessions*	
Sessions are client-led The therapist shows enthusiasm and interest towards the client The therapist is sympathetic towards the client, allowing him/her to ventilate their feelings The therapist is non-judgemental The therapist uses self-disclosure in moderation The therapist uses neutral tone, words and body language The therapist encourages the client to talk about their history/youth, encouraging them to reminisce The therapist encourages the client to talk about their family and friends The therapist encourages the patient to talk about any topic; this can include neutral topics such as hobbies, news, holidays, etc. or emotionally charged material	
*Things to avoid contaminating Talking control with therapeutic techniques*	
Setting an agenda for the session Trying to conceptualise the case Focusing on key problem areas Trying to conceptualise the case Applying specific ACT techniques Willingness to tolerate discomfort Mindfulness Cognitive fusion Conceptualising the problem in context Defining values Committed action Applying specific cognitive or behavioural techniques Asking for feedback about clients’ view/understanding of the session Trying to collaborate with clients to solve problems Trying to lead the client in a guided discovery to form new perspectives on problems Exploring underlying belief systems Encouraging healthy behaviours Setting assignments for out of therapy time	

### Analysis

All selected sessions were transcribed verbatim by DL (postgraduate student) and imported into NVivo (version 8.0), a software package used to aid storage, coding, and searching of data. DL was sufficiently neutral to the data as she had not been involved in the CanACT study nor the TC session. A thematic analysis was then conducted [20, 21]. Each transcript was first read in its entirety by DL (postgraduate student), who then conducted line by line coding to identify initial themes derived from the data. These initial themes were then analysed to identify higher order themes and their related sub-themes. To ensure validity and reliability, theme generation was also carried out independently by SD (a senior research nurse) and MA (senior research psychologist), who were familiar with the data and the research topic. The main findings were brought to the remaining team members (JL—health psychologist and MS—academic psychiatrist) for final confirmation.

## Results

### Participant characteristics

All participants were females with diagnoses of primary breast cancer with metastases. Four participants out of five were White British and one was Black British. Two out of five participants had higher educational degrees, while the other three had no qualifications above secondary level.

### Themes

Four main themes emerged from the data analysis. Participants used the sessions to release emotions, yet also avoided other difficult feelings and thoughts at times. The sessions were also used to focus on the positive aspects of individuals’ lives. Lastly, participants engaged in self-reflection on ways to overcome their difficulties.

1. Releasing feelings

TC sessions served as an opportunity for cathartic release. Participants discussed a variety of difficulties which included cancer-related issues as well as other personal or day-to-day struggles.

### Unleashing the weight of daily difficulties

The sessions served as a space in which participants were able to release frustration, anxiety or other emotions relating to their day-to-day struggles. For example, 4 out of 5 participants expressed feelings of being overwhelmed due to the accumulation of daily stresses and practical difficulties:

“*But yeah it’s just so many different people in and out and it’s just like oh god, when people phone me like that woman the other day…and I missed my acupuncture and I was quite angry*” Participant A, Session 4

Many participants also released feelings relating to their physical symptoms, issues with doctor appointments and other practical difficulties relating to their cancer.

### Allowing for the release of more difficult emotions

Aside from discussing day-to-day difficulties, participants (4 out of 5) often appeared to view the sessions as a safe space in which they could unleash more difficult feelings or emotions that had been burdening them for a while.

Many participants discussed how they felt with regards to the uncertainty of their illness. For instance, some participants revealed their distress regarding the worsening of their condition.

Participants also released feelings relating to how their illness was affecting other people around them, revealing, at times, possible feelings of guilt:

*“it is, very hard and to see that you can look at him (brother) and that he's very tired but he's just making sure that everything is, and not making you feel I tell him are you tired”* Participant B, Session 4

More difficult non-illness related emotions were also discussed. These often revolved around unresolved relationship or family difficulties, that had been burdening participants for a while. One participant repeatedly discussed her difficult relationship with her granddaughter:

*“I notice my relationship with my granddaughter is quite strained actually, we’re going away next weekend to…yeah I feel sad about it …”* Participant C, Session 4

A common difficulty that was expressed was the struggle of letting go of the past. For example, one participant discussed not being able to work anymore:

*“I’ve found it incredibly hard that adjustment of not working, I mean […] that adjustment ... I mean support work was a big part of my life… um so in that 6 months it was very difficult to sort of let go of my job”* Participant D, Session 1

The TC session even served as a space in which one participant was able to open up about suicidal feelings and engage in a more in-depth exploration of those thoughts. The degree to which emotions were discussed varied widely amongst participants. One participant was particularly open to discussing her feelings from the start, while three other participants opened up gradually about their difficult emotions as the sessions progressed. The participants brought up these concerns themselves rather than being prompted or asked by a therapist as they may do in therapy sessions. Many participants acknowledged the usefulness of being able to release their feelings. The TC was often viewed as a non-judgmental space which made it easier to discuss emotions, even for participants who didn’t often do so:

*“It’s nice to have done it (the therapy)…sometimes I've released my feelings which is the hardest thing for me to do…but you did get me at probably my worst time actually…but maybe that was good…”* Participant A, Session 8

One participant stood out in that she was not able to deal with many difficult emotions as she had done a substantial amount of therapeutical work in the past:

***“****You know I feel a bit like I wish I had an issue I could think of at the moment, so I could really try it out but at the moment its’ not like I haven’t had them in my life, but I think this whole process of having the cancer has taken through that, you know it’s been a process of dealing with that very very deeply”* Participant E, Session 5

2. Experiential avoidance

Even though the sessions were used as a means of cathartic release at times, participants also attempted to avoid certain thoughts, feelings, emotions and sensations, a behaviour known as experiential avoidance [21]. Domains that were avoided varied among participants, but emotions pertaining to their illness and prognosis were most often subject to experiential avoidance.

### Minimising

Difficult emotions or thoughts which related to the participants’ cancer were often minimised by participants and only discussed lightly A few participants engaged in laughter when discussing certain difficulties.

*“Because you always pain, if it’s not your neck then it’s your (laughs) you have always pain so that’s what they are trying to help s out to deal with the pain, sometimes you take the tablets but you know it can be effective for 45 minutes, 1 hour , 2 hour but you have to wait for 3 or more hours to take another one but uh…”* Participant B, Session 1

Others tried to maintain a positive mindset despite having to deal with a difficult situation, suggesting a possible attempt to deny negative feelings. A participant discussed plans with her friends:

*“I'm not eating—and I keep thinking I'm gonna be sick all the time, but yeah…I'm sure once I'm in that limo, I’ll be fine, got to get me in their first though—it’s quite high up apparently…mmm…but I will be in there somehow”* Participant A, Session 4

### Distancing

Participants also engaged in emotional avoidance by distancing themselves from their situation. In particular, some participants described their illnesses in a matter-of-fact way, possibly indicating a wish to disengage from the emotional charge associated with their situation:

*"Yes…like you never know, you never know, today the medicine is working tomorrow it’s not working anymore, and you are trying another drug and be helpful that every time you change your body will be able to support, to be in good condition to receive it because if you’re not well…”* Participant B, Session 4

### Escaping

Certain topics were completely avoided by some participants during the sessions, particularly in the beginning sessions. One participant briefly mentioned some difficulties such as a deterioration of symptoms but then did not seek to expand on the issue and shifted the conversation to other topics. One participant admitted to having engaged in cognitive avoidance:

*“Um…as you say, I haven’t been well for quite a while and I kept just…pretending I suppose that I was”* Participant A, Session 8

Participants also appeared to purposefully engage in a battle against their feelings as an attempt to keep difficult emotions at bay:

*“Because…I can be sort of…detached from emotion around it, although I wouldn’t want emotion around it all the time…I feel, I've got quite the lump in my throat now…thinking about it…oh god, go away lump”* Participant C, Session 4

3. Focusing on the positive

The TC sessions also served to engage with the more positive aspects of participants’ lives, cultivate gratitude and appreciate future sources of happiness.

### Discussing and recognising sources of joy and fulfilment

Sessions were used by some individuals as a way to take a step back and appreciate the sources of joy in their lives. Many participants discussed enjoyable moments they had experienced in previous days. For example, the following person described an enjoyable ice-skating outing she had enjoyed with her daughter a few days prior.

Participants discussed hobbies, and family and friends as important sources of positive emotions:

*“So, I'm starting to paint here, and that’s been a lot of fun and enjoyable”* Participant C, Session 4

Many of the participants also recognised the usefulness of their support system. In particular, the hospice services or cancer support groups were often discussed in a positive light. Positive talk also sometimes extended directly to their illness, where some participants reflected on the positive elements it had brought to their lives. One participant explained that having cancer had turned her life around and helped her get out of a depressive episode. Two other individuals mentioned the cancer as an opportunity to engage with new interests or activities:

*“I’m incredibly pleased that I’m having to do all these creative things that I absolutely love but I’ve always squeezed because there’s never been enough time, or I’ve never allowed enough time)”* Participant A, Session 1

### Engaging in future-oriented positive talk

The TC session also served to focus on the future, especially positive activities participants were looking forward to. Participants talked about looking forward to future activities such as family or friends’ gatherings or birthday celebrations. One participant talked about an upcoming spiritual event:

*“T: What was it um what was it how did you describe it last week?*

*P: Shaman*

*T: The shaman um… event*

*P: Yeah I’m really looking forward to that, that’s got me quite excited and uh - so its feels like some nice stuff it’s starting to happen, feels like coming out of the winter but with movement a bit more looking forward to - that is just a massive thing to look forward to…”* Participant E, Session 5

### Being grateful and appreciative

The TC were also used as an opportunity to foster feelings of gratitude. Many participants expressed being thankful to the help they received from their family members as well as medical staff. One participant acknowledged the efforts made by doctors whilst also recognising the challenges associated with treating cancer. Additionally, many participants were able to take a step back and recognise when they felt better physically, which often led to subsequent feelings of gratitude.

*“I’m getting better... it’s a pleasure that nobody knows if you were never in the situation yeah …small things now even to be able to go to the shower, and be there, wash yourself the way you like—you know it’s wonderful because you know how it is when you’re not able to do things… can’t begin to tell you and to feel now that you can wash yourself properly*” Participant B, Session 4

4. Reflecting on ways to handle adversity

Although to varying degrees, participants used the sessions to reflect on ways to handle difficulties.

### Reflecting on the importance of being psychologically flexible

It was acknowledged by some that to be able to manage adversity they would have to accept their thoughts and feelings. Some reflected on the idea that one can choose how to respond to difficulties and that uncertainty should be embraced rather than feared:

*“You know some days I feel, I am adjusting, I am getting on with this and then there’s bound to be moments when you just think, you just think argh, you know, and its completely natural and I know, no I’m not going to get of that completely and it’s about how you minimise that pushing against…. So, it is about …. That continual adapting to having sort of things to look froward to, having an aim within that uncertainty of, and within that acceptance of uncertainty…”* Participant D, Session 4

Mindfulness and meditation were mentioned as being helpful tools for three participants. One participant discussed the importance of acknowledging the fleeting nature of sensations and emotions:

*“And you see the thing with the pain was, because of the Vipassana I taught myself to experience things just as a vibration”* Participant E, Session 5

### Taking action to resolve difficulties

Participants also used the sessions as an opportunity to reflect on ways they could take action to resolve some of their difficulties. The importance of regular self-care was noted by some. One participant even mentioned that the TC sessions had served as a way of encouraging her to commit to self-care activities:

*“I thought it was quite interesting, everything I've said to you that I’ll do, I've done—which I can drag things out a bit, so there’s something positive”* Participant C Session 5

Two participants noted the importance of connection with people and finding the courage to reach out for help to the people around them. Two participants reflected on ways in which they could take action to solve family difficulties. For example, one participant reflected on her struggles with her granddaughter and decided she would talk to her honestly to try and solve their issues. Another common topic of reflection centered around the need for participants to alter their lifestyle habits in the aim of better managing their physical symptoms. Three participants mentioned the importance in engaging in regular pacing.

### Differences between participants

The participants presented with varying levels of self-reflection. Three participants engaged in occasional reflection, while another participant mainly used the sessions as a means of psychosocial reflection. She also planned the topics she wanted to discuss before the session to prioritise the areas she wanted to focus on. She also recognised that the TC provided her with a space to talk freely:

*“It’s (TC) incredibly helpful because it’s that space to think out loud and … if you have that space… it’s still sort of guidance of re-emerging to do those strategies and remember to focus on highlighting things that you want do differently”* Participant D, Session 8

One participant mainly discussed the reflection she had carried out in the past and how this had helped her get to a place of happiness and acceptance. She did not see the TC as being useful for her as she did not have many issues she was struggling with. She also noted that her friends already provided her with a space in which she could reflect on potential difficulties and perceived the therapist’s approach as not being ‘fully’ genuine (i.e., paid to be there).

## Discussion

This study is the first to explore the content of talking control sessions using a qualitative approach to determine if is an appropriate control. It also provides an opportunity to explore what people chose to talk about. When provided with a space to talk, individuals with advanced cancer are willing to discuss their feelings and difficulties openly, albeit to varying degrees. The confidential and non-judgmental aspects of the chat as well as not seeing the therapist after the sessions may have also encouraged the disclosure of personal issues. Sharing thoughts and feelings with someone who did not know them personally nor their history, appeared to help them divulge and express themselves fully. Expression of feelings appears to explain why TC was deemed beneficial by some participants. Being able to discuss feelings and emotions freely has been identified as an important need for people with cancer [22, 23]. Indeed, befriending has been considered useful by people with cancer partly because of the opportunity to discuss sensitive topics and feelings [24, 25], but befriending does not control for advice and problem solving that may be delivered by a trained friend who may have gained experience on how to manage various aspects of problems associated with having cancer. The disclosure of suicidal feelings by one participant also demonstrates that TC was seen as a safe space for cathartic release which could be used as a way of monitoring suicidal ideation and managing risk.

The tendency of participants to engage in experiential avoidance and focusing on the positive during the TC may partly be explained by evidence showing that cancer patients can prefer discussing difficult topics with their partners, family members or friends, rather than with a health care practitioner [26]. This is in line with the finding of one participant who indicated that the TC was not useful to them, as they already had sufficient external support. Yet, according to Osse and colleagues [27], some people with cancer may also feel reluctant to discuss difficulties with partners or family members for fear of burdening them. It is possible TC may be more beneficial for those without sufficient support from family and friends.

People with cancer used TC sessions to undertake reflection on ways to overcome adversity, although to what extent varied. This process was never encouraged by the therapist but initiated by the participants themselves. The people who did engage in reflection identified both acceptance and taking action as key to overcoming their struggles. This is consistent with evidence suggesting that Acceptance and Commitment Therapy is effective for decreasing psychological distress in patients with cancer [28, 29]. It is important to note that the TC’s use as a means of reflection was heterogeneous. The participants who engaged in much deeper self-reflection had experience of engaging with therapy in the past. Therefore, it could be that previous exposure to therapy leads participants to use TC to continue applying learned therapeutic techniques.

Overall, it appears there may be some therapeutic benefits of TC beyond the common effects of therapy, which is largely dependent on the patient as opposed to the therapist. The TC may be used as a space to find solutions to problems and therefore not control only for the common effects of therapy dependent on whether reflection is considered an effect of the therapy that is being tested or not. Another instance where this occurs is through relaxation, which has been shown to lead people to engage in self-reflection and perhaps indirect behavioural change, yet it is commonly used for controlling for common effects of therapies in RCTs [11].

This study was the first of its kind to provide an in-depth analysis of the content of a Talking Control. Thematic analysis was able to capture the complexity and richness of the data. The first author (DL) was not involved in the original study, which allowed for a more neutral position and analysis of the data. The findings were explored in an iterative process between the team members ensuring strong rigour. The data was naturalistic between the therapist and the patients and therefore was not influenced by an interviewer, making the conclusions stronger. A larger sample size may help ensure saturation of the data and enabled a more representative sample by including male participants and range of cancer diagnoses.

Using TC as a control condition should be done so with caution and monitored throughout to ensure specific therapeutic effects are not being used. The TC analysis also revealed that people with advanced cancer had other issues they wished to discuss that were important to them such as familial difficulties. This might suggest that interventions for individuals with advanced cancer should focus on overall life-difficulties and adopt a more person-centred approach to therapy. Further research is needed to explore whether the TC translates into improved outcomes or whether self-reflection without guidance could exacerbate negative thoughts and feelings.

From a clinical perspective, our findings suggest people benefit for a space to express how they feel and be heard by an empathic person who is at the same time detached from their immediate loved ones. TC has many characteristics of Rogerian Psychotherapy but differs in that it does not offer problem solving and needs not be undertaken by a psychologist. Therefore, the TC offers a potential alternative mental health intervention for those who are reluctant to undergo a more action-oriented therapy.

## Conclusions

People with advanced cancer have approached TC sessions as a safe place in which they can express feelings for example from smaller daily concerns to deeper-rooted difficulties. Many participants may also engage in emotional and cognitive avoidance about some topics, particularly those pertaining to their cancer and prognosis. Additionally, participants used the TC as an opportunity to focus on the positive. Avoiding cancer-related topics and engaging in such ‘normal talk’ could be important for generating feelings of hope. Whilst the therapists were discouraged to engage in reflective processes, the findings show it is not possible to prevent people from self-reflecting on ways to overcome difficulties. Our research has helped identify what people with advanced cancer wish to talk about which may then provide an insight into therapeutic approaches.

## Data Availability

The data that support the findings of this study are available from the corresponding author upon reasonable request.
